# Cryogenic 3D Printing of w/o Pickering Emulsions Containing Bifunctional Drugs for Producing Hierarchically Porous Bone Tissue Engineering Scaffolds with Antibacterial Capability

**DOI:** 10.3390/ijms23179722

**Published:** 2022-08-27

**Authors:** Xinliang Ye, Zhi He, Yuming Liu, Xiaoying Liu, Rouye He, Ganhang Deng, Ziqing Peng, Jiayu Liu, Zicai Luo, Xiaoling He, Xiang Wang, Jing Wu, Xiaowei Huang, Jingying Zhang, Chong Wang

**Affiliations:** 1 School of Mechanical Engineering, Dongguan University of Technology, 1 Daxue Road, Songshan Lake, Dongguan 523808, China; 2 Second Clinical School of Medicine, Guangdong Medical University, Dongguan 523710, China; 3 Division of Science and Technology, Dongguan University of Technology, Dongguan 523808, China; 4 Guangdong-Hong Kong-Macao Joint Laboratory for Neutron Scattering Science and Technology, Songshan Lake, Dongguan 523808, China

**Keywords:** bone tissue engineering, cryogenic 3D printing, Pickering emulsion, osteogenesis, antibacterial

## Abstract

How to fabricate bone tissue engineering scaffolds with excellent antibacterial and bone regeneration ability has attracted increasing attention. Herein, we produced a hierarchical porous β-tricalcium phosphate (β-TCP)/poly(lactic-co-glycolic acid)-polycaprolactone composite bone tissue engineering scaffold containing tetracycline hydrochloride (TCH) through a micro-extrusion-based cryogenic 3D printing of Pickering emulsion inks, in which the hydrophobic silica (h-SiO_2_) nanoparticles were used as emulsifiers to stabilize composite Pickering emulsion inks. Hierarchically porous scaffolds with desirable antibacterial properties and bone-forming ability were obtained. Grid scaffolds with a macroscopic pore size of 250.03 ± 75.88 μm and a large number of secondary micropores with a diameter of 24.70 ± 15.56 μm can be fabricated through cryogenic 3D printing, followed by freeze-drying treatment, whereas the grid structure of scaffolds printed or dried at room temperature was discontinuous, and fewer micropores could be observed on the strut surface. Moreover, the impartment of β-TCP in scaffolds changed the shape and density of the micropores but endowed the scaffold with better osteoconductivity. Scaffolds loaded with TCH had excellent antibacterial properties and could effectively promote the adhesion, expansion, proliferation, and osteogenic differentiation of rat bone marrow-derived mesenchymal stem cells afterward. The scaffolds loaded with TCH could realize the strategy to “kill bacteria first, then induce osteogenesis”. Such hierarchically porous scaffolds with abundant micropores, excellent antibacterial property, and improved bone-forming ability display great prospects in treating bone defects with infection.

## 1. Introduction

Bone tissue engineering, which combines cells and growth factors with a highly porous biomimetic bone tissue engineering scaffold, has been increasingly used to induce bone regeneration [1]. Since bacterial infections can easily occur on artificial bone implants, providing bone tissue engineering scaffolds with excellent antibacterial property to prevent post-implantation infection is of great importance [2]. A desirable bone tissue engineering scaffold not only provides temporary mechanical support during the tissue regeneration process but also provides cells with a suitable microenvironment for anchoring, adhesion, proliferation, and differentiation [3]. Therefore, it is particularly crucial to endow the scaffold with appropriate shape and structure properties to elicit favorable biological responses. Towards bone tissue engineering scaffolds, a hierarchical porous structure is beneficial to cell migration, adhesion, and nutrient transfer/metabolic waste discharge [4].

Among the various strategies that have been used to fabricate porous scaffolds, the casting of Pickering emulsions has shown great potential in making scaffolds with very high porosity. Pickering emulsions refer to the use of solid particles as emulsion stabilizers that aggregate at the interface of two immiscible liquids (i.e., oil phase and water phase) to stabilize droplets and prevent their coalescence [5,6]. In a typical w/o Pickering emulsion, both discontinuous water droplets and continuous organic solutions occupy a very high portion, while the polymer matrix and solid particulate emulsifier only hold a small portion. Once the organic solvents (i.e., oil phase) and the water (i.e., aqueous phase) which normally exceed 75% of the total emulsion volume, are removed to obtain scaffolds, an interconnected porous structure with very high porosity can be acquired [6]. However, the simple casting of w/o Pickering emulsions, followed by solvent evaporation, can only produce microporous scaffolds with a pore size below 100 μm, making it difficult to enable cell infiltration, which requires a much larger pore size [7,8,9,10]. Three-dimensional printing is particularly suitable for building tissue engineering scaffolds with personalized shapes and tailored porous structures to create a biomimetic structural environment that can facilitate cell infiltration, enhance vascularization, and promote tissue formation [11,12]. By using traditional 3D printing techniques such as fused deposition modeling (FDM), selective laser sintering (SLS), and digital light projection (DLP), polymeric scaffolds with a macroscopic pore size of 200–600 μm can be easily produced. Such a pore size could facilitate cell crawling, nutrient transfer, metabolite clearance, and neovascularization [13,14,15]. Nevertheless, these scaffolds’ lack of secondary micropores (pore size: 1–100 μm) on the strut surface show insufficient microtopographic cues, which are important for fast cell adhesion and spreading. Three-dimensional printing w/o polymeric Pickering emulsions can be employed to fabricate tissue engineering scaffolds with both macroscopic grid structures (pore size: hundreds of microns) and secondary micropores (pore size: <100 μm) on the strut surface. Yang et al. [7] formulated a Pickering emulsion consisting of water and PCL-PLLA/DCM solution by using hydrophobic silica nanoparticles (h-SiO_2_) as emulsifiers. The Pickering emulsion was used as printing inks to fabricate porous scaffolds via micro-extrusion-based 3D printing. Compared with other 3D printed bone tissue engineering scaffolds [16,17], scaffolds 3D printed from Pickering emulsions exhibited a hierarchical porous structure with very high porosity. However, such scaffolds printed from polyester-based Pickering emulsion inks without the delivery of any biologically active agent such as bioactive ceramics and osteogenic drugs lack a bone-forming ability. Given that calcium and phosphate ions generated along with β-TCP degradation can help to promote the mineralization of bone marrow mesenchymal stem cells and osteoblasts [13], and the 3D printing of Pickering emulsion inks containing a certain amount of β-TCP nanoparticles would endow scaffolds with better osteoconductivity.

Towards the antibacterial effect, tetracycline hydrochloride (TCH), a broad-spectrum antibiotic with a good bactericidal effect by preventing the growth of bacterial peptide chains and protein synthesis, has gained increasing attention. TCH inhibits bacterial growth at low concentrations and kills bacteria at high concentrations [18]. Additionally, TCH has also been reported to affect bone metabolism by affecting the function of osteoclasts. In addition, TCH was found to promote the activity and proliferation of osteoblasts and rat bone marrow-derived mesenchymal stem cells (rBMSCs) and enhance the expression of osteogenic markers such as osteocalcin, type I collagen, and osteocalcin at low concentrations [19]. Therefore, a certain amount of TCH can be loaded into the porous scaffold to treat bone defects with infection through a “kill bacteria first, then promote osteogenesis” strategy, in which the burst TCH release can prevent the adhesion of bacteria on the scaffold surface and kill the peripheral bacteria, while a slow but steady TCH release with a low concentration can promote the proliferation and osteogenic differentiation of rBMSCs in a long-term manner.

In this study, micro-extrusion-based cryogenic 3D printing was employed to fabricate TCH-loaded β-tricalcium phosphate/poly(lactic-*co*-glycolic acid)-poly(caprolactone) (β-TCP/PLGA-PCL) antibacterial bone tissue engineering scaffolds with interconnected porous structures by using w/o composite Pickering emulsion as printing inks. The effects of the contents of β-TCP and h-SiO_2_ (emulsifier), the printing temperatures and drying temperatures on the structure of the scaffolds, were systematically investigated. The in vitro TCH release and scaffold degradation were also studied. The antibacterial study and in vitro cell culture study suggested that the scaffolds had excellent antibacterial properties and an improved bone-forming ability. Our study provides a feasible scheme for constructing a hierarchical porous bifunctional bone tissue engineering scaffold to treat bone defects with infection.

## 2. Results

### 2.1. Scaffold Design

**Figure 1:**
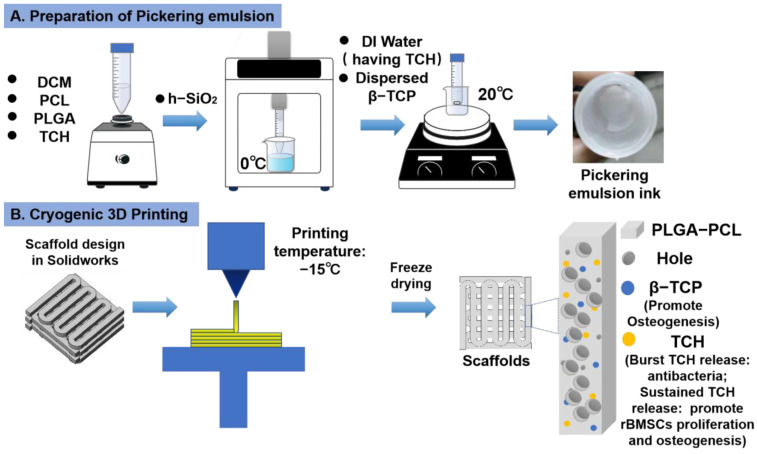
Schematic illustration of Pickering emulsion ink formulation and 3D printing of drug-loaded scaffolds. (**A**) The formulation of Pickering emulsion inks. (**B**) Cryogenic 3D printing of hierarchically porous bone tissue engineering scaffolds loaded with TCH.

To produce a bone tissue engineering scaffold with a biomimetic porous structure, excellent antibacterial capability, and improved bone-forming ability, in this study, dual-delivery Pickering emulsion inks with a high volume internal water phase were formulated (Figure 1A). The printing inks were then subjected to a micro = extrusion-based cryogenic 3D printing to obtain predesigned 3D products, followed by a freeze drying treatment (Figure 1B). PLGA-PCL polymers were used as the basic material to construct the 3D grid patterns and acted as the delivery carrier of osteoconductive TCP particles and TCH drugs, while the presence of DCM and DI water in the printing inks and their removal after cryogenic 3D printing were responsible for the formation of micropores on the struts.

### 2.2. Characterization of Pickering Emulsions

**Figure 2:**
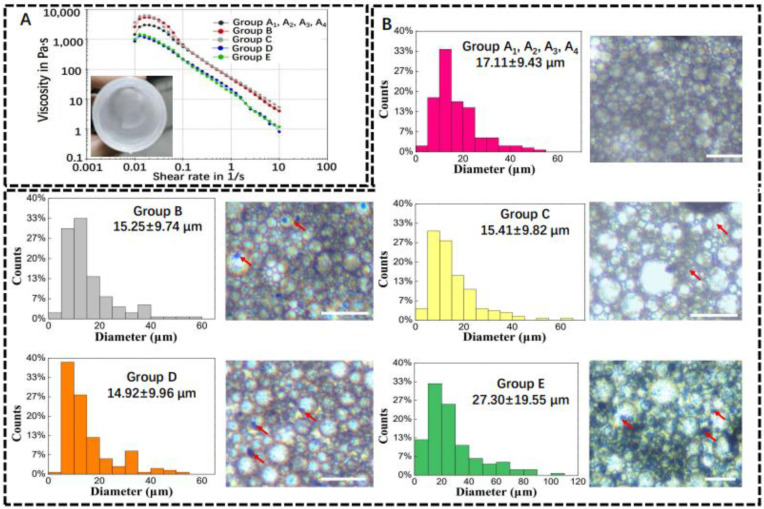
Physical properties of w/o Pickering emulsion inks. (**A**) Viscosity–shear rate plot and milky white Pickering emulsion ink. (**B**) Average diameter of water droplets in Pickering emulsion inks and their representative images. Red arrows represent β-TCP (*n* = 150). Scale bar is 100 μm.

Prior to 3D printing, the viscosity of the Pickering emulsion inks was measured using a viscometer. As shown in Figure 2A, the emulsion inks in all groups showed a decrease in viscosity with an increasing shear rate, which indicated that all Pickering emulsions had shear thinning properties. Viscous w/o Pickering emulsion inks with a milky white state could be successfully formulated. The as-formulated w/o emulsion inks were stable enough for micro-extrusion-based 3D printing. The structure of Pickering emulsion inks with varied compositions was observed using optical microscopy. It can be seen from Figure 2B that the spherical water droplets in different groups had an average diameter of 17.11 ± 9.43 μm (Group A_1,_ A_2_, A_3_, and A_4_); 15.25 ± 9.74 μm (Group B); 15.41 ± 9.82 μm (Group C); 14.92 ± 9.96 μm (Group D); and 27.30 ± 19.55 μm (Group E), respectively. β-TCP agglomerates (red arrows in Figure 2B) were dispersed in the emulsions and could affect the stability of the water/oil interface.

### 2.3. Characterization of Scaffolds Printed from Pickering Emulsion Inks

**Figure 3:**
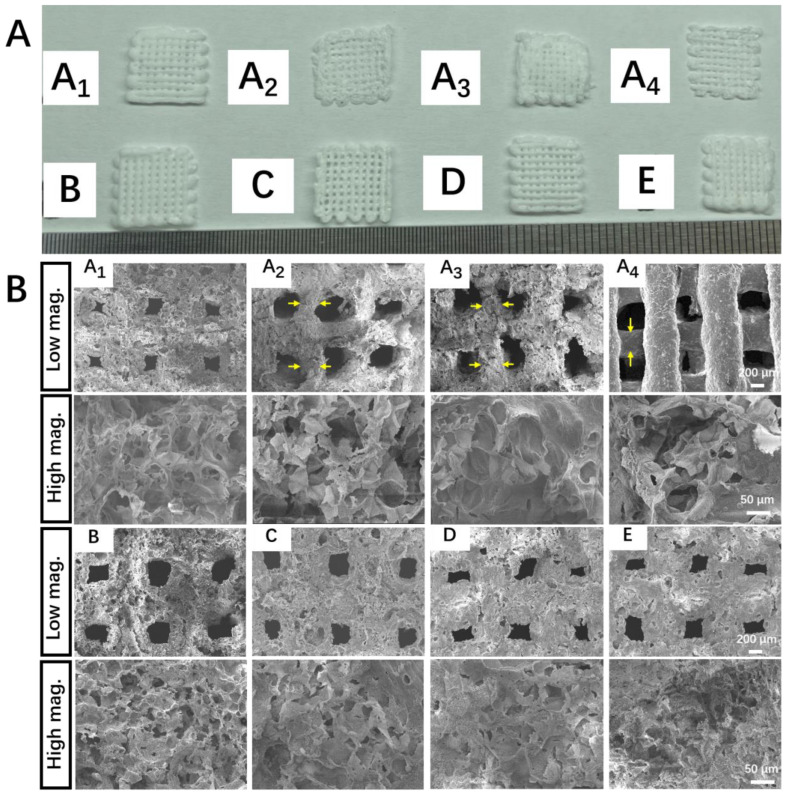
Morphology of porous scaffolds made from w/o Pickering emulsion inks. (**A**) Digital images of printed scaffolds. (**B**) SEM micrographs of different scaffolds (yellow arrows represent the thinning of the struts). A1 to E represent the top view of scaffolds made through A1 to E Pickering emulsion inks at low magnification (50×) and high magnification (350×), respectively.

**Figure 4:**
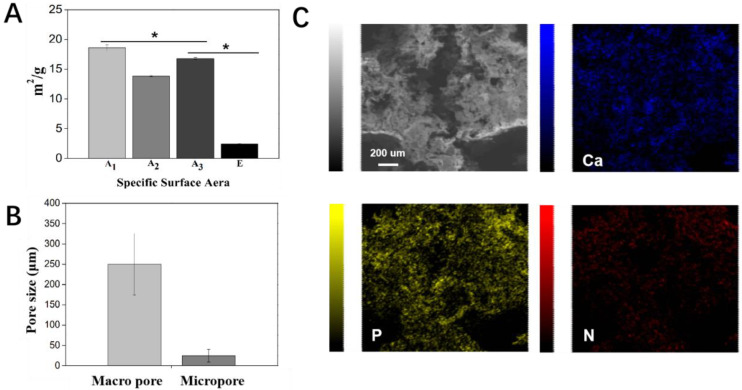
Physical characterization of scaffolds. (**A**) Specific surface area of Group A_1_, A_2_, and A_3_ and E. (**B**) Pore size of the macroscopic pore and micropore on struts in Group E. (**C**) EDS-X elemental mapping of Ca, P, and N on scaffolds in Group E-TCH_1_. * *p* < 0.05.

A comparative study was first conducted to investigate the effect of the printing temperature and drying temperature on the structure of printed scaffolds without the addition of β-TCP particles. For each emulsion ink formula, at least 20 scaffolds were printed. All the scaffolds were printed under the same working parameters. A1 scaffolds, which were printed and dried at low temperatures, had continuous grid patterns, and abundant secondary micropores were observed on the struts. The dimensions of the A1 scaffolds were identical to that of the CAD model (i.e., strut diameter: 600 μm; the distance between the center lines of the two paralleled struts was 1000 μm), showing a high reproducibility. In comparison, the struts in Group A_2_, A_3_, and A_4_ that involved room temperature printing and/or room temperature drying appeared to shrink to a certain degree and even collapse, showing a lower reproducibility. Meanwhile, much fewer micropores were observed on the struts of these scaffolds (Figure 3B). These results suggest cryogenic 3D printing and vacuum freeze drying could facilitate the formation of more micropores on struts and lead to a more complete scaffold structure. It is also found that adding more β-TCP particles into the Pickering emulsion inks could endow the printed scaffolds with clearer outlines, and this trend could be attributed to the increased viscosity brought by higher β-TCP contents. However, a reduction in the number of micropores on struts could be observed in Groups B–E (Figure 3B). As a result, Group E, which involved the highest content of β-TCP, showed the lowest specific surface area (Figure 4A). Additionally, we evaluated the macroscopic pore and secondary micropore size of Group E, which were 250.03 ± 57.88 and 24.70 ± 15.56 μm, respectively (Figure 4B). Through EDX spectroscopy, the presence of β-TCP on the surfaces of the scaffolds in Group E was confirmed (Figure 4C).

### 2.4. In Vitro TCH Release Behaviour

**Figure 5:**
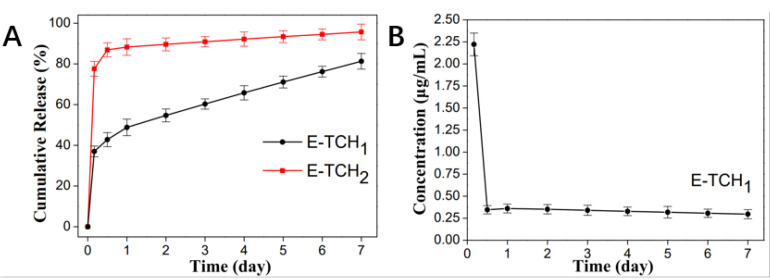
In vitro release behavior of TCH in different drug-loaded scaffolds. (**A**) In vitro drug release of E-TCH_1_ and E-TCH_2_ in a 7-day release period. (**B**) The residual TCH concentration at each time point.

In our study, a strategy of “kill bacteria first, then induce osteogenesis” can be achieved by loading the appropriate concentrations of TCH in the oil phase and water phase, respectively. The cumulative release curve is shown in Figure 5A. The scaffolds exhibited a burst TCH release up to 101.65 ± 2.51 μg/mL in 4 h, which could realize the purpose of killing bacteria quickly. Afterwards, a slow but steady TCH release from 0.3 ± 0.05 μg/mL to 2.22 ± 0.13 μg/mL was achieved in 7 days, in which the TCH concentrations (0.25–8 μg/mL) could contribute to the proliferation and osteogenic differentiation of rBMSCs (Figure 5B) [20].

### 2.5. Antibacterial Property of Porous Scaffolds

**Figure 6:**
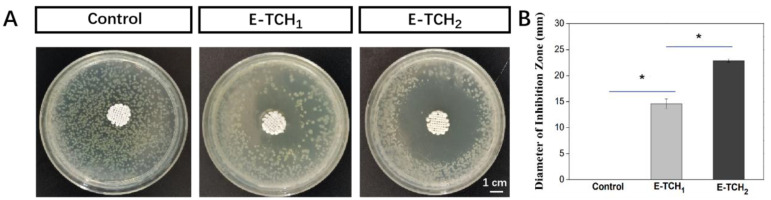
Antibacterial properties of porous scaffolds: (**A**,**B**) inhibition zone induced by different scaffolds after 24-h culture; (**B**) average diameter of the inhibition zone. * *p* < 0.05.

**Figure 7:**
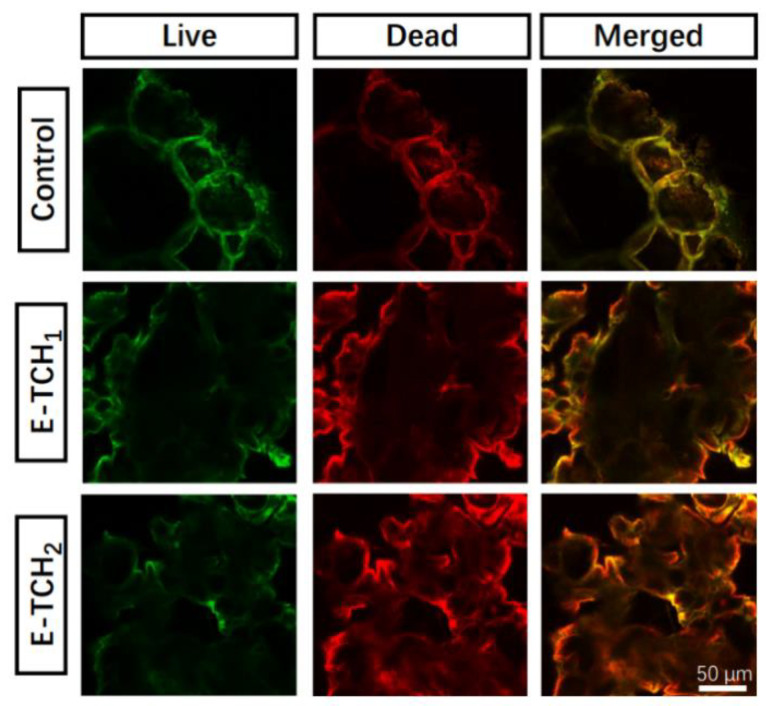
Live and dead staining of *S. aureus* cultured on different scaffolds for 4 h.

To combat bacterial infections, biomaterials should have effective antibacterial capabilities [21]. The antibacterial activity of scaffolds was investigated in vitro against Gram-positive bacterium (*Staphylococcus aureus*), which are the common bacterium that causes bone infections [21]. The antibacterial properties of the scaffolds were verified by agar diffusion testing. As expected, there was no inhibition zone around the control scaffolds but obvious inhibition zones with radii of 12.3 ± 1.2–22.9 ± 0.3 mm were found around the scaffolds loaded with a varied amount of TCH (Figure 6A,B). Live and dead staining was also employed to show the antibacterial property of scaffolds (Figure 7). After 4 h of incubation of bacteria on the porous scaffolds, the red fluorescence (dead bacteria) and green fluorescence (live bacteria) showed the same intensity in the control group. In comparison, the intensity of the red fluorescence signal was enhanced in the E-TCH_1_ group, and the strongest red fluorescence intensity was obtained in the E-TCH_2_ group (Figure 7), indicating that the TCH-loaded scaffolds possessed excellent antibacterial properties.

### 2.6. In Vitro Viability and Osteogenic Differentiation of rBMSCs on Drug Loaded Scaffolds

**Figure 8:**
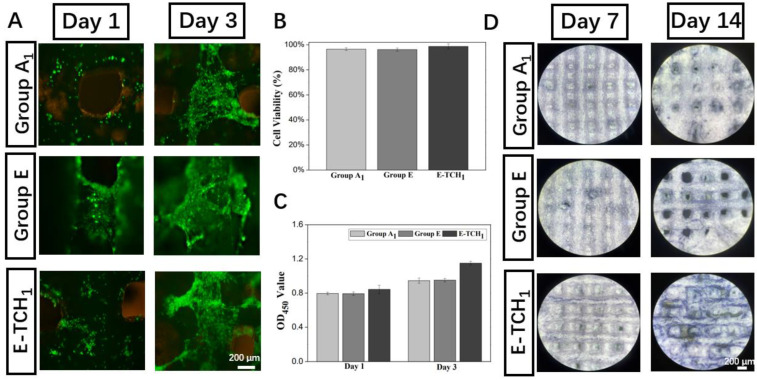
Biological performance of scaffolds: (**A**) live and dead staining images at day 1 and day 3, (**B**) cell viability after 1 day of culture, (**C**) cell proliferation on scaffolds in 3 days, and (**D**) ALP staining of rBSMCs on different scaffolds after 7- and 14 days of culture.

Biocompatibility is always considered a key factor for the application of tissue engineering scaffolds in the biomedical field. To test the cytocompatibility, the scaffolds cultured with rBMSCs were subjected to a cell viability test and live and dead staining (Figure 8A,B). A large number of viable cells (color in green) and only a few dead cells (color in red) were observed on all scaffolds, indicating that drug delivery scaffolds made through the cryogenic 3D printing of Pickering emulsion inks were a favorable platform for cell seeding. Then, cell proliferation was detected by the CCK8 assay. The OD_450_ value showed that the proliferative activity of rBMSCs increased gradually with the increasing culture time (Figure 8C). More importantly, the OD_450_ value of TCH-loaded scaffolds was the highest among all the groups. Afterwards, the bone-forming ability of the drug delivery scaffolds was evaluated in vitro. The expression of ALP, a marker of early osteogenic differentiation, can be used to indicate the osteogenesis potent scaffolds. After 7–14 days of culture (Figure 8D), the ALP expression (color in purple) in both Group E and Group E-TCH_1_ were higher than that in Group A_1_, and a higher ALP expression (larger area and darker purple color) could be observed in Group E-TCH_1_ after 14 days of culture, suggesting that scaffolds with sustained TCH release had the ability to induce the osteogenic differentiation of rBMSCs and can be used as a bifunctional material to treat a bone defect with infection.

## 3. Discussion

In clinical orthopedics, bacterial infection after trauma, bone tumor-related tissue resection, etc. often leads to the failure of bone repair and/or bone regeneration. The formation of biofilms will promote the formation of chronic wounds, making it very difficult to effectively treat the bone defect. So far, regenerating bone tissue in infection regions is still challenging. Bioactive bone tissue engineering scaffolds with high porosity and hierarchically interconnected pores are designed to promote proper cellular responses such as cell migration, proliferation, and osteogenic differentiation and improved tissue regeneration [7]. Towards the treatment of bone defects in the infection region, a bone tissue engineering scaffold with suitable antibacterial capability is necessary [22,23,24,25]. In this study, a hierarchically porous scaffold with excellent antibacterial capability and osteogenic activity was made through a cryogenic 3D printing of a Pickering emulsion containing β-TCP nanoparticles and TCH.

Since the structure of Pickering emulsion is crucial to the printability of Pickering emulsion inks and the spatial structure of the printed scaffold, the contents of h-SiO_2_ and β-TCP should be carefully tuned. It is known that the size of the solid particles used to stabilize the water/oil interface of Pickering emulsions is usually small (basically smaller than 3 μm), and the addition of oversize particles will reduce the overall stability of the Pickering emulsions. Meanwhile, Pickering emulsions with higher stability normally have a smaller water droplet size [26,27]. As the size of β-TCP used in the current investigation was much larger than 3 μm (β-TCP is obtained after passing through a screen with a pore size of 70 μm), most β-TCP can only be dispersed either in water droplets or a continuous oil phase.

The printing temperature and drying temperature significantly affected the scaffold structure. When the scaffolds were printed and dried at room temperature, the molecular chains of PCL-PLGA matrices were still freely movable in DCM solvent. Hence, the volatilization of DCM would cause the inward movement of the molecular chains of PLGA and PCL matrices to the central region of the strut, forming dried struts with a smaller diameter. If the movement rates of molecular chains of PCL-PLGA matrices to the central region are not consistent everywhere, the diameters of struts become uneven, and scaffold collapse could also occur. Additionally, as water droplets have a much higher boiling point than DCM (100 °C vs. 39 °C), the strut thinning induced by the volatilization of DCM would squeeze out many water droplets that were originally located in the struts/on the strut surface, hence showing struts with less micropores. In comparison, when the scaffolds were printed at a low temperature (i.e., −15 °C) and freeze dried at −50 °C, the molecular chains of PCL-PLGA matrices of the as-printed “wet” struts were constantly frozen, and water droplets embedded in the struts were also frozen into ice microparticles. In such cases, the removal of the organic solvent (i.e., DCM) and water phase (i.e., ice particles) through freeze drying would not affect the distribution of the molecular chains of PCL-PLGA in the scaffold matrix, hence forming struts with a uniform diameter and leaving numerous micro-holes/pores in the struts/on the strut surface. With the above consideration, whether the molecular chains of the PCL-PLGA matrix and water droplets were frozen or not during the printing and drying procedure is the predominant factor to influence the microstructure of the scaffolds. Since the freeze drying machine used in this study had a working temperature of −50 °C, we only selected −50 °C as the freeze drying temperature to remove DCM and water from the cryogenic 3D printed scaffolds.

It is known that if the bacterial infection is not effectively controlled in the early implantation stages, the formation of biofilms will exacerbate the infection. The adhesion between bacteria and implants is the first and most important stage of this process [19]. Loading antibiotics in the implant can solve the problem. Tetracycline antibiotics have been used clinically for over decades and are active against a variety of Gram-positive and Gram-negative bacteria [16]. These antibiotics can cause various metabolic disorders in bacteria, including inhibiting protein synthesis, nucleic acid synthesis, oxidic phosphate enzymes, and various oxidation and fermentation reactions [28]. The minimum bacteriostatic concentration (MIC) is considered to represent the inherent activity of each antibacterial agent. The MIC50 and MIC90 of tetracycline in vitro are 0.25–2 μg/mL and 32 μg/mL for Gram-positive bacteria (Staphylococcus aureus) and 2 μg/mL and 64 μg/mL for Gram-negative bacteria (Escherichia coli), respectively [29,30]. In our design, the Pickering emulsion has a high concentration of TCH in the water phase and a low concentration in the oil phase. The bacteria are killed effectively in 4 h by a burst release of TCH. Moreover, the concentration of the post-release TCH can also achieve the purpose of becoming bacteriostatic (Figure 5). Our scaffolds exhibit excellent antibacterial properties of short-term sterilization and long-term bacteriostasis.

In terms of bone tissue regeneration, scaffolds with hierarchical porous structures are beneficial for the anchoring, spreading, and the osteogenic differentiation of rBMSCs. Group E has a macroscopic pore size of 250.03 ± 75.88 μm and contains numerous secondary micropores of 24.70 ± 15.56 μm in size, which meets the needs of bone tissue engineering (i.e., macroscopic pore size: 200–600 μm [13]). Furthermore, the loaded bioactive ceramics, β-TCP, can rapidly degrade to generate calcium and phosphate ions that contribute to the differentiation of rBMSCs and subsequent mineralization [31]. Our results showed that the presence of β-TCP in hierarchically porous scaffolds significantly improved rBMSC differentiation and cell mineralization compared to the control group. As a common antibacterial drug, TCH not only has broad-spectrum antibacterial properties but also has the effect of promoting the proliferation of rBMSCs at an appropriate concentration (0.25–8 μg/mL) [19,20,32]. Moreover, TCH can affect bone metabolism by affecting the function of osteoclasts. For example, osteoclasts are induced to undergo apoptosis, reduce fold boundary areas and acid production, and selectively inhibit ontogenesis [33]. Therefore, dual functional TCH with both excellent antibacteria capability and pro-osteoblast proliferation ability can be used as a bifunctional drug to enhance bone tissue engineering. The results indicate that we have realized the strategy of “kill bacteria first, then induce osteogenesis”. Our hierarchical porous scaffolds with the dual delivery of β-TCP and TCH meet the antibacterial properties required for implants while promoting the proliferation and differentiation of rBMSCs.

## 4. Materials and Methods

### 4.1. Materials

Polycaprolactone (PCL) with a molecular weight of 100,000 and phosphate buffer solution (PBS) were supplied by Sigma-Aldrich (St. Louis, MO, USA). Poly(lactic-co-glycolic acid) (PLGA) with a molecular weight of 260,000 was provided by Jinan Daigang Biotechnology Ltd., Jinan, Shandong, China. β-tricalcium phosphate (β-TCP) was purchased from Shanghai Bio-lu Biomaterials (China). Hydrophobic silica nanoparticles (h-SiO_2_) were obtained from Wacker Chemie (Burghausen, Germany). Tetracycline hydrochloride (TCH) was purchased from Macklin (Shanghai, China). Dichloromethane (DCM) was purchased from Shanghai Aladdin Ltd. Deionized (DI) water was produced from a DI water producer (Sartorius arium mini plus, Goettingen, Germany).

### 4.2. Formulation of Pickering Emulsion Inks

Given that h-SiO_2_ is a nondegradable nanoparticle, to reduce the toxicity brought by h-SiO_2_ [34], the content of β-TCP in Pickering emulsions was increased, while the content of h-SiO_2_ was reduced as much as possible. Pickering emulsion was prepared following a protocol in a previous study [7]. Briefly, 0.3 g PCL and 0.3 g PLGA were first dissolved in 10 mL of DCM. Next, a certain amount of h-SiO_2_ nanoparticles (0.075, 0.125, and 0.25 g) was added to the polymer solution, followed by ultrasonication for 10 min at 5 °C. Then, 23.3 mL of DI water and a certain amount of β-TCP nanoparticles (0, 0.125, 0.175, 0.25, 0.375, 0.5, and 0.625 g) were dispersed in the PCL-PLGA/DCM solution loaded with h-SiO_2_ and magnetically stirred at room temperature for 30 min at 1000 rpm, thereby obtaining Pickering emulsion with a water phase/oil phase ratio of 3:7. Table 1 details the ingredients of different groups of Pickering emulsion inks. The emulsion preparation process is shown in Figure 1A.

### 4.3. Fabrication of Porous Scaffolds

The CAD model with a wood crib structure was designed using SolidWorks (USA) and converted to a STL file format. A self-developed low-temperature 3D printer comprising a X-Y-Z motion platform, an extrusion system, and a refrigerated box was used to fabricate scaffolds. A 20-mL syringe was used to load w/o Pickering emulsion inks and further loaded in the low-temperature 3D printer. The piston of the syringe was driven by a screw at a feeding rate of 0.002 mm/s to extrude printing inks out of a V-shaped nozzle (inner diameter: 0.6 mm) to draw a continuous pattern layer-by-layer. A refrigerated box was used to stabilize the printing temperature, which was set as −15 °C. A typical CAD scaffold model had a 5-layer structure, and each layer had 10 paralleled cylindrical struts. The distance between the center lines of the two paralleled struts was 1000 μm, and the intersection angle of the struts at the adjacent layers was 90°. The layer thickness of the scaffolds was set as 0.25 mm, and the printing speed was set as 5 mm/s. After low-temperature 3D printing, the as-fabricated scaffolds were subjected to freeze drying treatment to obtain dried scaffolds. Scaffolds printed (i.e., Group A_3_ and A_4_) and dried (i.e., Group A_2_ and A_4_) at room temperature were used as control groups. All dried scaffolds subjected to antibacteria study and cell culture were sterilized beforehand by immersing in 75% ethanol (*v*/*v*, ethanol/DI water = 0.75) for 5 min, followed by 5 min of rinsing in PBS for 3 times.

### 4.4. Physical Characterization of Pickering Emulsions and Porous Scaffolds

The viscosity of Pickering emulsions was measured by a rheometer (MCR 702 MultiDrive, Anton Paar, Graz, Austria) at 20 °C, equipped with stainless-steel plates (diameter: 40 mm) with a 1-mm gap between the plates. Viscosity tests were performed at shear rates ranging from 0.01 to 10 s^−1^. The structure of Pickering emulsion inks was observed under an inverted fluorescence microscope (Eclipse TE2000-U, Nikon, Tokyo, Japan). The diameter of the droplets and the size of the pores were analyzed by ImageJ (Version 1.53K, National Institutes of Health, Bethesda, MD, USA). The macroscopic morphological images of the porous scaffold were captured by a digital camera (iPhone 12), and the microscopic morphology of the scaffolds was observed using an optical microscope and a SEM (JSM-IT500A, JEOL Ltd., Tokyo, Japan). The specific surface area of different scaffolds was measured by ASAP 2460 Version 2.02. The pretreatment temperature of the scaffold was 40 °C, and the pretreatment time was 16 h.

### 4.5. In Vitro Release Behavior of Tetracycline Hydrochloride (TCH)

It is reported that a sustained TCH release can promote the proliferation and differentiation of rBMSCs [19,32], while a high TCH concentration can effectively kill bacteria [35]. In the current study, to produce drug-loaded scaffolds with a sustained TCH release profile (designated as E-TCH_1_), 1 mg of TCH was dispersed in 10 mL DCM. In contrast, to produce drug-loaded scaffolds with both burst TCH release and sustained TCH release (designated as E-TCH_2_), 1mg of TCH was dispersed in 10 mL DCM, and 23.3 mg of TCH was dissolved in 23.3 mL DI water. The rest of the contents and fabrication process of the TCH-loaded scaffold was the same as that of Group E. The absorption concentration standard curve of TCH, and the release kinetics of TCH from the scaffold were determined by a microplate reader at 372 nm (TECAN-Spark, Shanghai, China). Release the experimental samples in a constant temperature shaker (Zhengrong Instrument, Jintan, China) at 37 °C with a vibration speed of 50 r/min. Three control groups were set up at each time point, and 200 μL of the solution was withdrawn at fixed times.

### 4.6. Evaluation of Antibacterial Properties of Porous Scaffolds

*Staphylococcus aureus* (a Gram-positive bacterium) was used as a model bacterium to verify the antibacterial activity of the scaffolds. After adjusting the concentration of *S. aureus* to 1 × 10^6^ CFU/mL, the bacterial suspension was spread on the surface of agar for inoculation. Next, the scaffold was placed in the center of the agar plate to coculture with *S. aureus* for 24 h at 37 °C and photographed to record the inhibition zone. In the live and dead staining, *S. aureus* was cultured until the turbidity of the bacterial suspension was about 0.8. Subsequently, the collected bacteria were diluted 100 times. The resuspended bacteria were cocultured with scaffolds on 24-well plates for 4 h for live and dead staining.

### 4.7. Cell Culture

Rat bone marrow mesenchymal stem cells (rBMSCs) were cultured in Dulbecco’s modified Eagle’s medium (DMEM, Gibco, New York, NY, USA) containing 10% fetal bovine serum (Gibco, New York, NY, USA), 100 U/mL penicillin–streptomycin, and 2 mM L-glutamic acid (Invitrogen, Carlsbad, CA, USA). The culture plates containing rBMSCs and culture medium were placed in an incubator with an ambient temperature of 37 °C and a volume fraction of 5% CO_2_ saturated humidity. The medium was changed every 36 h. After adding the sterilized scaffolds in the wells of 24-well plates, 0.2 mL of rBMSC cell solution with a concentration of 1 × 10^6^ cell/mL was seeded on each scaffold. After culturing for 3 h, 1.8 mL of DMEM was added afterward.

### 4.8. rBMSCs Proliferation and Osteogenic Differentiation on Scaffolds

The cytocompatibility of the scaffolds was investigated by staining with a live–dead staining kit (Molecular Probes, Eugene, OR, USA) at 1 and 3 days, in which live and dead cells were stained green and red, respectively. Scaffolds were placed in DMEM containing 4 μM EthD-1 and 2 μM calcein-AM for 15 min in a humidified incubator (37 °C, 5% CO_2_), and then, photos were taken at 2 time points using a fluorescence microscope (Nikon Eclipse TE2000-U inverted microscope, Japan). The proliferation of rBMSCs on the porous scaffolds was measured using the CCK8 (Dojindo, Kumamoto City, Japan) proliferation assay at day 1 and after 3 days of culture, respectively. After culturing scaffolds for 7 and 14 days, an ALP staining kit (Puhe Biomedical Technology, Wuxi, China) was used to study the osteogenic differentiation of rBMSCs.

### 4.9. Statistical Analysis

All statistical analyses were performed using the SPSS software (version 18). Numerical data were presented as the mean value ± standard deviation (S.D.). For the statistical comparisons, one-way analysis of variance (ANOVA) with the Student’s *t*-test was applied. *p* < 0.05 (*) was considered to be statistically significant, in which (*) was used to indicate the significant differences in the histological images.

## 5. Conclusions

In this study, highly porous bone tissue engineering scaffolds with effective antibacterial property and excellent osteogenesis capability were produced through the cryogenic 3D printing of β-TCP and TCH loaded w/o composite Pickering emulsion inks and the subsequent freeze drying treatment. The printed struts had a microporous surface with a very high surface area-to-volume ratio and, hence, could be used as an excellent delivery vehicle for antibacterial drugs. Since the loading of high dosage of TCH in the water phase of w/o Pickering emulsion inks could lead to a burst TCH release with a high concentration from the scaffolds, the effective elimination of *S. aureus* bacteria in a short time period can be achieved, hence meeting the early antibacteria needs after scaffold implantation. The slow but sustained release of TCH not only inhibited the growth of *S. aureus* in the long term but also promoted the proliferation of rBMSCs. Moreover, the osteogenic differentiation of rBMSCs was promoted in the presence of β-TCP and a sustained release of TCH with a low concentration.

## Figures and Tables

**Table 1:** Compositions of different Pickering emulsion inks.

Group Designation	DCM (mL)	DI Water (mL)	PCL (g)	PLGA (g)	TCP (g)	h-SiO_2_ (g)	Printing Temperature (°C)	Drying Temperature (°C)
A_1_	10	23.3	0.3	0.3	0	0.25	−15	−50
A_2_					0	0.25	−15	20
A_3_					0	0.25	20	−50
A_4_					0	0.25	20	20
B					0.125	0.125	−15	−50
C					0.25	0.125	−15	−50
D					0.375	0.125	−15	−50
E					0.5	0.125	−15	−50

## References

[1] Lai J.H., Wang C., Chen S.S., Liu C.Y., Huang J.W., Pan Y., Xie Y.C., Wang M. (2022). Low temperature hybrid 3D printing of hierarchically porous bone tissue engineering scaffolds with in situ delivery of osteogenic peptide and mesenchymal stem cells. Biofabrication.

[2] Zhao C.Q., Liu W.Y., Zhu M., Wu C.T., Zhu Y.F. (2022). Bioceramic-based scaffolds with antibacterial function for bone tissue engineering: A review. Bioact. Mater..

[3] Dhandayuthapani B., Yoshida Y., Maekawa T., Kumar D.S. (2011). Polymeric scaffolds in tissue engineering application: A review. Int. J. Polym. Sci..

[4] Hollister S.J. (2005). Porous scaffold design for tissue engineering. Nat. Mater..

[5] Gonzalez Ortiz D., Pochat-Bohatier C., Cambedouzou J., Bechelany M., Miele P. (2020). Current Trends in Pickering Emulsions: Particle Morphology and Applications. Engineering.

[6] Yang Y.Q., Fang Z.W., Chen X., Zhang W.W., Xie Y.M., Chen Y.H., Liu Z.G., Yuan W.E. (2017). An overview of pickering emulsions: Solid-particle materials, classification, morphology, and applications. Front. Pharmacol..

[7] Yang T., Hu Y., Wang C.Y., Binks B.P. (2017). Fabrication of Hierarchical Macroporous Biocompatible Scaffolds by Combining Pickering High Internal Phase Emulsion Templates with Three-Dimensional Printing. ACS Appl. Mater. Interfaces.

[8] Hu Y., Gu X.Y., Yang Y., Huang J., Hu M., Chen W., Tong Z., Wang C.Y. (2014). Facile fabrication of poly(l -Lactic Acid)-Grafted hydroxyapatite/poly(lactic-co-glycolic Acid) scaffolds by pickering high internal phase emulsion templates. ACS Appl. Mater. Interfaces.

[9] Liu H., Wang C.Y. (2014). Chitosan scaffolds for recyclable adsorption of Cu(ii) ions. RSC Adv..

[10] Li Z., Ngai T. (2010). Macroporous polymer from core-shell particle-stabilized pickering emulsions. Langmuir.

[11] Wong K.V., Hernandez A. (2012). A Review of Additive Manufacturing. ISRN Mech. Eng..

[12] Richards D.J., Tan Y., Jia J., Yao H., Mei Y. (2013). 3D printing for tissue engineering. Isr. J. Chem..

[13] Rao S.H., Harini B., Shadamarshan R.P.K., Balagangadharan K., Selvamurugan N. (2018). Natural and synthetic polymers/bioceramics/bioactive compounds-mediated cell signalling in bone tissue engineering. Int. J. Biol. Macromol..

[14] Marjan B. (2020). Challenges on optimization of 3D-printed bone scaffolds. Biomed. Eng. OnLine.

[15] María V., Eduardo R. (2011). Bioceramics: From Bone Regeneration to Cancer Nanomedicine. Adv. Mater..

[16] Wang C., Ye X.Y., Zhao Y.T., Bai L., He Z., Tong Q., Xie X.Q., Zhu H.R., Cai D.Z., Zhou Y. (2020). Cryogenic 3D printing of porous scaffolds for in situ delivery of 2D black phosphorus nanosheets, doxorubicin hydrochloride and osteogenic peptide for treating tumor resection-induced bone defects. Biofabrication.

[17] Wang C., Huang W., Zhou Y., He L.B., He Z., Chen Z.L., He X., Tian S., Liao J.M., Lu B.H. (2020). 3D printing of bone tissue engineering scaffolds. Bioact. Mater..

[18] Hash J.H., Wishnick M., Miller P.A. (1964). On the Mode of Action of the Tetracycline Antibiotics in Staphylococcus. J. Biol. Chem..

[19] Zhang J., Xue S.L., Luo Y., Zhi W. (2017). Tetracycline hydrochloride promotes rat bone marrow mesenchymal stem cells proliferation in vitro. Chin. J. Tissue Eng. Res..

[20] Chen X., Yang Z.M., Jie H.Q., Li F.S. (2005). Biological effect of WO-1 on human embryonic osteoblasts. Chin. J. Reparative Reconstr. Surg..

[21] Chen Z.Y., Gao S., Zhang Y.W., Zhou R.B., Zhou F. (2021). Antibacterial biomaterials in bone tissue engineering. J. Mater. Chem. B.

[22] Ribeiro M., Monteiro F.J., Ferraz M.P. (2012). Infection of orthopedic implants with emphasis on bacterial adhesion process and techniques used in studying bacterial-material interactions. Biomatter.

[23] Schierholz J.M., Beuth J. (2001). Implant infections: A haven for opportunistic bacteria. J. Hosp. Infect..

[24] Sánchez-Salcedo S., Colilla M., Izquierdo-Barba I., Vallet-Regí M. (2016). Preventing bacterial adhesion on scaffolds for bone tissue engineering. Int. J. Bioprinting.

[25] Versey Z., da Cruz Nizer W.S., Russell E., Zigic S., DeZeeuw K.G., Marek J.E., Overhage J., Cassol E. (2021). Biofilm-Innate Immune Interface: Contribution to Chronic Wound Formation. Front. Immunol..

[26] Wu J., Ma G.H. (2016). Recent Studies of Pickering Emulsions: Particles Make the Difference. Small.

[27] Low L.E., Siva S.P., Ho Y.K., Chan E.S., Tey B.T. (2020). Recent advances of characterization techniques for the formation, physical properties and stability of Pickering emulsion. Adv. Colloid Interface Sci..

[28] Chopra I., Roberts M. (2001). Tetracycline Antibiotics: Mode of Action, Applications, Molecular Biology, and Epidemiology of Bacterial Resistance. Microbiol. Mol. Biol. Rev..

[29] Bunick C.G., Keri J., Tanaka S.K., Furey N., Damiani G., Johnson J.L., Grada A. (2021). Antibacterial mechanisms and efficacy of sarecycline in animal models of infection and inflammation. Antibiotics.

[30] Zhanel G., Critchley I., Lin L.Y., Alvandi N. (2019). Microbiological profile of sarecycline, a novel targeted spectrum tetracycline for the treatment of acne vulgaris. Antimicrob. Agents Chemother..

[31] Bose S., Roy M., Bandyopadhyay A. (2012). Recent advances in bone tissue engineering scaffolds. Trends Biotechnol..

[32] Zhang J., Chen X.H., Li L., Li X.H., Zhi W., Zhu H.M., Deng L. (2010). Effect of Tetracycline-HCl sustained release microspheres on the activity of rat osteoblast in vitro. West China Med. J..

[33] Vernillo A.T., Rifkin B.R. (1998). Effects of tetracyclines on bone metabolism. Adv. Dent. Res..

[34] Napierska D., Thomassen L.C.J., Lison D., Martens J.A., Hoet P.H. (2010). The nanosilica hazard: Another variable entity. Part. Fibre Toxicol..

[35] Dashti A., Ready D., Salih V., Knowles J.C., Barralet J.E., Wilson M., Donos N., Nazhat S.N. (2010). In vitro antibacterial efficacy of tetracycline hydrochloride adsorbed onto Bio-Oss^®^ bone graft. J. Biomed. Mater. Res.—Part B Appl. Biomater..

